# Supplementary material for: Combining Metabolomics and Experimental Evolution Reveals Key Mechanisms Underlying Longevity Differences in Laboratory Evolved Drosophila melanogaster Populations
Source: Int J Mol Sci. 2022 Jan 19;23(3):1067. doi: 10.3390/ijms23031067 (PMC8835531; doi:10.3390/ijms23031067)
Supplement: Supplementary file 1 [file ijms-23-01067-s001.zip › ijms-1526526-supplementary.pdf]

International Journal of
Molecular Sciences

MDPI

Supplementary Figures

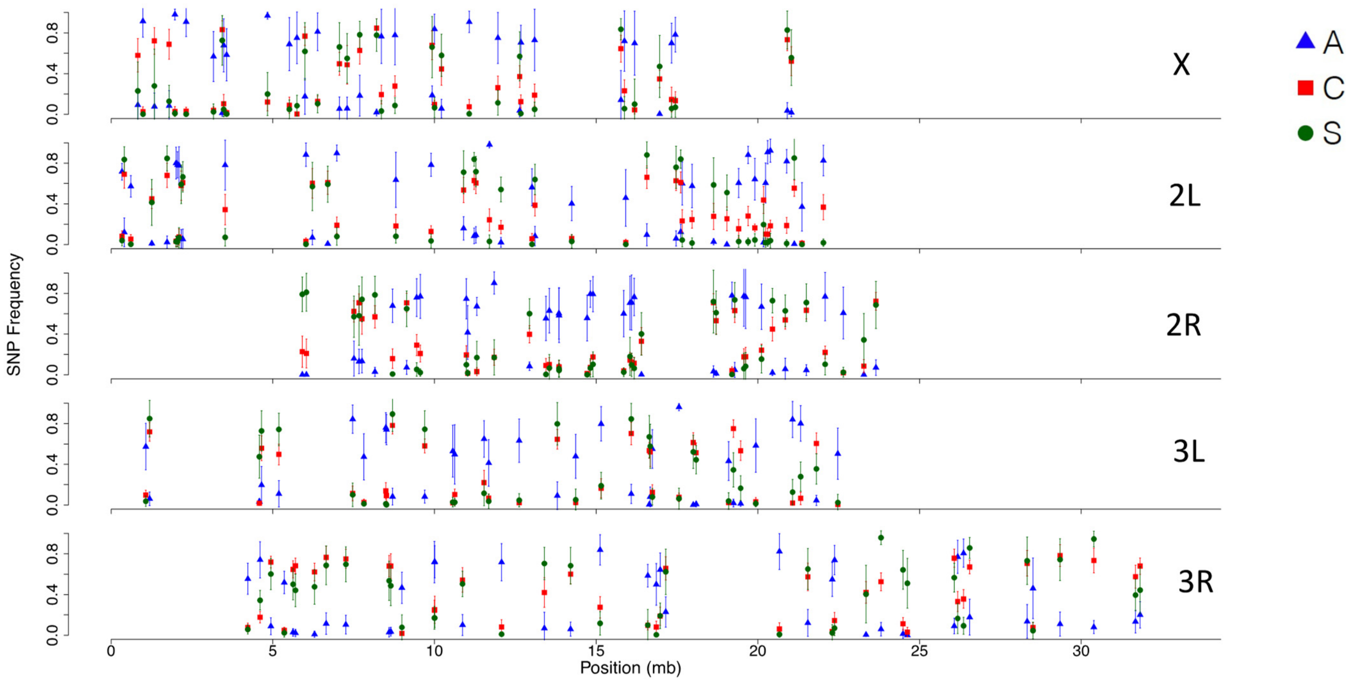

Supplementary Figure S1. Mean SNP frequencies in the A, C, and S populations at sites that best predict patterns of candidate metabolites differentiation based on FLAM analysis. Panels represent the major chromosome arms and error bars are based on standard deviation.

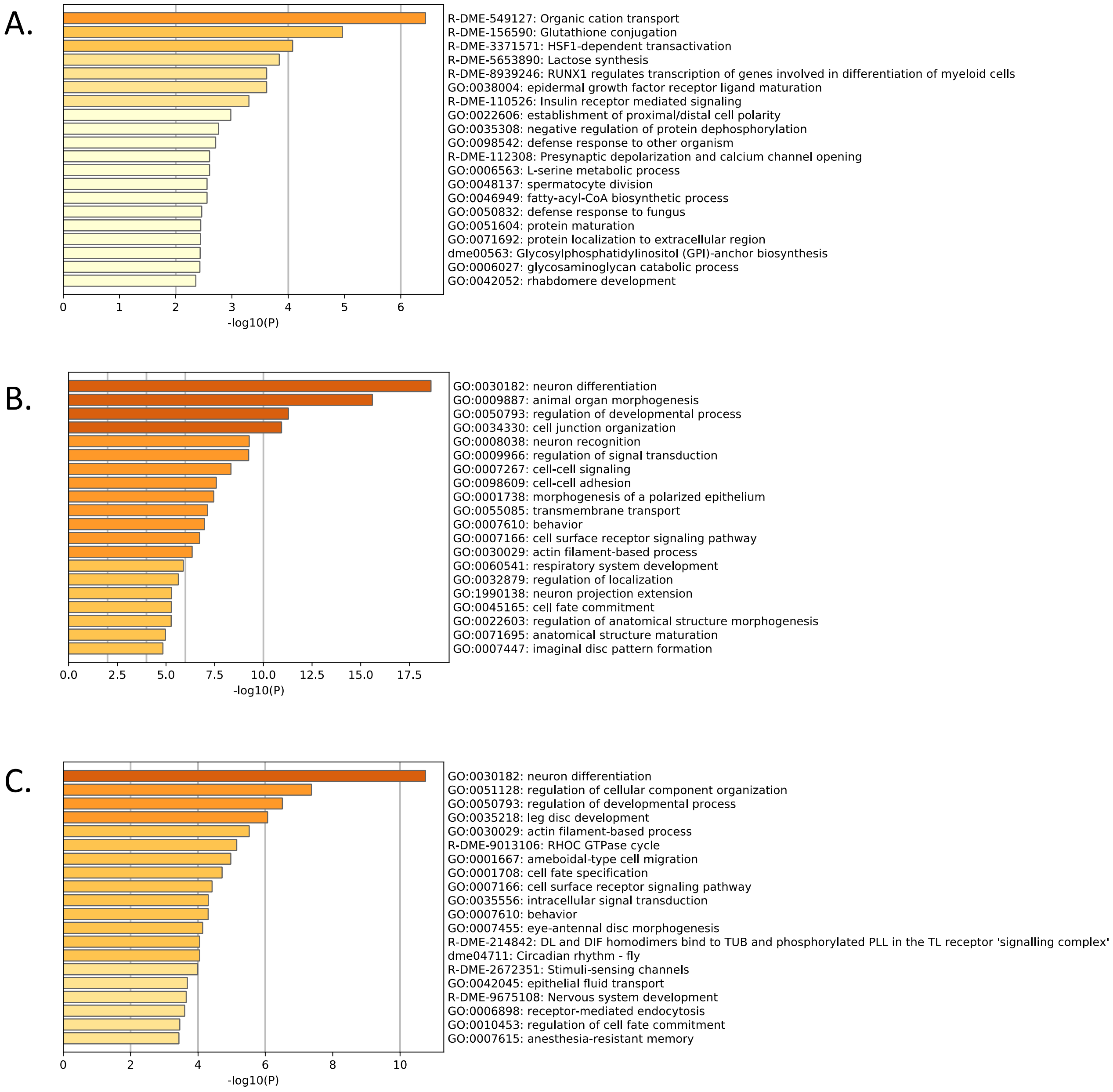

Supplementary Figure S2. Heatmap of top 20 enriched GO clusters based on candidate longevity genes identified Remolina et al. 2012 (A), Carnes et al. 2015 (B), and Fabian et al. 2018 (C).

Supplementary Table S1. Quantification of metabolites in selected pathways from A, C, and S populations.

Metabolite	Number of Markers	Best Predictors
2-Hydroxybutyrate	4	2R _ 5844860; 2R _ 5963523; 3R _ 17006052; 3R _ 24487864
Fructose 6-phosphate	9	2R _ 14819199; 2R _ 14900655; 2R _ 18626758; 2R _ 20113582; 2R _ 21499617; 2R _ 23652889; 3L _ 16641565; 3R _ 16596451; X _ 6386704
beta-Alanine	12	2L _ 23234007; 2R _ 11027172; 2R _ 23278066; 3L _ 12618507; 3L _ 18090746; 3R _ 26362279; 3R _ 31822448; 3R _ 8648671; X _ 10217007; X _ 5747134; X _ 7299556; X _ 9924933
Glutamate	7	2L _ 19044057; 2R _ 21040098; 2R _ 9450843; 2R _ 9560497; 3R _ 24224131; 3R _ 24277382; X _ 15765284
Linolenic.acid	2	2R _ 12245832; 3L _ 3578076
Pantothenic.acid	4	2L _ 21126984; 2L _ 6700190; X _ 21036769; X _ 8209696
Glucose	4	2L _ 23234007; 3L _ 10626748; X _ 7687854; X _ 8777397
Ornithine	36	2L _ 10901218; 2L _ 12057191; 2L _ 3914630; 2L _ 406826; 2L _ 4185298; 2L _ 5984306; 2L _ 6224506; 2L _ 6980913; 2R _ 12939777; 2R _ 19198631; 2R _ 19285534; 2R _ 19932231; 2R _ 20450592; 3L _ 11532540; 3L _ 1189331; 3L _ 16084023; 3L _ 16641565; 3L _ 16738689; 3L _ 19244899; 3L _ 21323066; 3R _ 13022947; 3R _ 14210034; 3R _ 14302644; 3R _ 15121370; 3R _ 22371707; 3R _ 23807788; 3R _ 26550590; 3R _ 5625410; 3R _ 9999967; X _ 12634649; X _ 17450484; X _ 20910407; X _ 2320629; X _ 3167341; X _ 3482143; X _ 7060032
Glucose 6-phosphate	15	2R _ 16175094; 2R _ 16399091; 3L _ 13797475; 3L _ 18002930; 3L _ 19467979; 3L _ 21074097; 3L _ 21810579; 3R _ 10865975; 3R _ 26072981; 3R _ 4226699; 3R _ 4607431; 3R _ 5699934; 3R _ 6288920; 3R _ 8596385; X _ 3435806
Ribose	14	2L _ 20240634; 2L _ 2052345; 2L _ 3914630; 2L _ 6023618; 2L _ 754101; 2R _ 14719900; 2R _ 15853015; 2R _ 22642400; 3L _ 17561209; 3L _ 7817961; 3R _ 22301692; 3R _ 8992279; X _ 1973406; X _ 985091
Isoleucine	8	2L _ 1257106; 2L _ 20388629; 2L _ 2100095; 2R _ 10985897; 2R _ 7756408; 3L _ 7468486; 3R _ 29345975; 3R _ 4226699
Cysteine	10	2L _ 15916054; 2R _ 13849488; 2R _ 19566863; 2R _ 20846246; 2R _ 7508246; 2R _ 7665341; 3L _ 4650854; 3R _ 13396497; 3R _ 21547008; X _ 5511643

O-Phosphoethanolamine	25	2L _ 11225773; 2L _ 11280594; 2L _ 12057191; 2L _ 19905372; 2L _ 20301910; 2L _ 21364394; 2L _ 3527183; 2L _ 608680; 2L _ 8807469; 2R _ 11310365; 2R _ 13446096; 2R _ 13549455; 2R _ 5912550; 2R _ 6039610; 2R _ 9450843; 3L _ 10626748; 3L _ 14897413; 3L _ 16672160; 3L _ 8496585; 3L _ 9699354; 3R _ 24661183; 3R _ 30391842; 3R _ 4939622; X _ 11037787; X _ 11073347
Methionine	16	2L _ 13015587; 2L _ 13778828; 2L _ 17964831; 2L _ 20890099; 2R _ 22642400; 2R _ 8701989; 3L _ 11675911; 3L _ 14367581; 3L _ 8514087; 3R _ 12075148; 3R _ 20665943; 3R _ 25319810; 3R _ 5353381; X _ 13096496; X _ 16187747; X _ 8357568
Alanine	8	2L _ 14244503; 2L _ 16568269; 2R _ 13446096; 2R _ 22079715; 3L _ 19095912; 3L _ 5706848; X _ 11959931; X _ 17331721
Sedoheptulose 7-phosphate	11	2L _ 10901218; 2L _ 18632655; 2L _ 2167427; 2L _ 2218568; 2L _ 6224506; 2R _ 11846148; 2R _ 18710535; 2R _ 9140422; 3R _ 26179104; X _ 16961375; X _ 17450484
Histidine	9	2L _ 19407741; 2L _ 9893331; 2R _ 13849488; 2R _ 16046270; 3R _ 16858683; 3R _ 16979165; 3R _ 28327214; 3R _ 28507162; X _ 4835869
Methylmalonate	17	2L _ 11698817; 2L _ 17471254; 2L _ 17616379; 2L _ 17661704; 2L _ 19700425; 2L _ 2007186; 2L _ 336508; 2R _ 19622055; 2R _ 8157865; 3L _ 10566061; 3L _ 1071510; 3L _ 19813679; 3L _ 19936198; 3L _ 22474229; 3R _ 12075148; X _ 10000615; X _ 15874461
6-Phosphogluconate	10	2L _ 20175490; 3L _ 15152339; 3L _ 4584101; 3R _ 17151320; 3R _ 31679316; 3R _ 6650170; 3R _ 7259621; X _ 1791080; X _ 5994262; X _ 826048
Leucine	9	2L _ 1257106; 2L _ 20388629; 2L _ 2100095; 2R _ 16095574; 2R _ 6602890; 3R _ 10006316; 3R _ 23346094; 3R _ 24624760; X _ 1336466
Valine	19	2L _ 13107278; 2L _ 1731476; 2L _ 17661704; 2L _ 19700425; 2L _ 2100095; 2L _ 22027671; 2L _ 336508; 2R _ 10985897; 2R _ 13851138; 2R _ 19566863; 2R _ 8157865; 3L _ 1071510; 3L _ 22474229; 3L _ 5186537; 3L _ 8700555; 3R _ 14302644; X _ 10000615; X _ 12671176; X _ 3572210
L-Palmitoylcarnitine	11	2L _ 16012303; 2R _ 19566863; 2R _ 21530194; 3L _ 19095912; 3R _ 7911715; 3R _ 8781885; X _ 10125408; X _ 11737115; X _ 12995444; X _ 17352572; X _ 4634413
Stearoylcarnitine	10	2L _ 15800266; 2R _ 10895586; 2R _ 15195669; 2R _ 15335980; 3L _ 1115073; 3L _ 13441554; 3L _ 9609278; 3L _ 9703056; 3L _ 9966898; 3R _ 16130109
AMP	5	2L _ 20175490; 2R _ 14534436; 3R _ 6713117; X _ 249692; X _ 583399
Adenosine	11	2L _ 13623290; 2L _ 20125315; 2R _ 22262026; 2R _ 9605780; 3L _ 10626748; 3L _ 14489349; 3L _ 4584101; 3L _ 6688225; X _ 20014124; X _ 22448494; X _ 826048
FAD	22	2L _ 15079742; 2L _ 20552741; 2L _ 23007041; 2L _ 7303509; 2R _ 14819199; 2R _ 19424171; 3L _ 1031031; 3L _ 12674306; 3L _ 15903205; 3L _ 16002593; 3L _ 5290175; 3L _ 8212366; 3R _ 11670253; 3R _ 11919624;

		3R _ 16534108; 3R _ 17151320; 3R _ 25849857; 3R _ 7857361; X _ 10843588; X _ 19511063; X _ 2320629; X _ 2655239
Carnitine	12	2L _ 12150199; 2L _ 247688; 2L _ 6525595; 2R _ 15945624; 2R _ 20571485; 2R _ 23596755; 2R _ 23609823; 2R _ 8261483; 3L _ 1455155; 3R _ 10865975; 3R _ 31104987; 3R _ 5271641
Uracil	12	2L _ 11594159; 2L _ 15916054; 2L _ 247688; 2R _ 14819199; 2R _ 19424171; 2R _ 19566863; 2R _ 19622055; 3L _ 8212366; 3R _ 16234148; 3R _ 22738649; X _ 10843588; X _ 11643203
CMP	3	2R _ 14580314; 3R _ 24277382; X _ 1973406
GMP	7	2R _ 14580314; 2R _ 23107421; 2R _ 6250639; 3L _ 4743069; 3R _ 24224131; 3R _ 24277382; 3R _ 24371268

Supplementary Table S2. Quantification of high energy state metabolites in selected pathways from A, C, and S populations.

Chromosome	Number of Candidate Regions
2L	64
2R	66
3L	56
3R	66
X	54

Supplementary Table S3. A complete list of candidate genes based on FLAM results linking genomic regions to metabolite differentiation.

FlyBase Gene ID	Gene Symbol	GO terms
FBgn0266557	kis	GO:1902683 regulation of receptor localization to synapse;GO:1902685 positive regulation of receptor localization to synapse;GO:0097120 receptor localization to synapse
FBgn0265149	lncRNA:CR44218	NA
FBgn0051637	CG31637	GO:0006044 N-acetylglucosamine metabolic process;GO:1901071 glucosamine-containing compound metabolic process;GO:0006040 amino sugar metabolic process
FBgn0000320	eya	GO:0016576 histone dephosphorylation;GO:0070285 pigment cell development;GO:1901099 negative regulation of signal transduction in absence of ligand
FBgn0015400	kek2	NA
FBgn0267327	Acp33A	GO:0045434 negative regulation of female receptivity, post-mating;GO:0007621 negative regulation of female receptivity;GO:0046008 regulation of female receptivity, post-mating
FBgn0051760	CG31760	GO:0007186 G protein-coupled receptor signaling pathway;GO:0007165 signal transduction;GO:0023052 signaling
FBgn0259984	kuz	GO:0035386 regulation of Roundabout signaling pathway;GO:0035385 Roundabout signaling pathway;GO:0061320 pericardial nephrocyte differentiation
FBgn0028879	CG15270	GO:0006821 chloride transport;GO:0015698 inorganic anion transport;GO:0006820 anion transport
FBgn0028645	beat-Ib	GO:0007157 heterophilic cell-cell adhesion via plasma membrane cell adhesion molecules;GO:0098742 cell-cell adhesion via plasma-membrane adhesion molecules;GO:0008045 motor neuron axon guidance
FBgn0032577	CG13244	NA
FBgn0028644	beat-Ic	GO:0007415 defasciculation of motor neuron axon;GO:0007414 axonal defasciculation;GO:0007157 heterophilic cell-cell adhesion via plasma membrane cell adhesion molecules
FBgn0284409	lncRNA:TS1	GO:0007283 spermatogenesis;GO:0048232 male gamete generation;GO:0003006 developmental process involved in reproduction
FBgn0250907	Cht10	GO:0006032 chitin catabolic process;GO:0046348 amino sugar catabolic process;GO:1901072 glucosamine-containing compound catabolic process
FBgn0050369	CG30369	GO:0008150 biological_process

FBgn0033278	CG14759	GO:0008150 biological_process
FBgn0033279	CG2291	GO:0008150 biological_process
FBgn0033280	CG12126	GO:0008150 biological_process
FBgn0050376	CG30376	GO:0008150 biological_process
FBgn0033434	CG1902	GO:0120009 intermembrane lipid transfer;GO:0006869 lipid transport;GO:0010876 lipid localization
FBgn0265312	lncRNA:CR44285	NA
FBgn0050000	GstT1	GO:0006749 glutathione metabolic process;GO:0006575 cellular modified amino acid metabolic process;GO:0006790 sulfur compound metabolic process
FBgn0050005	GstT2	GO:0006749 glutathione metabolic process;GO:0006575 cellular modified amino acid metabolic process;GO:0006790 sulfur compound metabolic process
FBgn0033437	CG12926	GO:0120009 intermembrane lipid transfer;GO:0006869 lipid transport;GO:0010876 lipid localization
FBgn0050020	CG30020	GO:0045944 positive regulation of transcription by RNA polymerase II;GO:0045893 positive regulation of transcription, DNA-templated;GO:1902680 positive regulation of RNA biosynthetic process
FBgn0017414	cag	NA
FBgn0033569	CG12942	GO:0006355 regulation of transcription, DNA-templated;GO:1903506 regulation of nucleic acid-templated transcription;GO:2001141 regulation of RNA biosynthetic process
FBgn0033570	ND-B14	GO:0010257 NADH dehydrogenase complex assembly;GO:0032981 mitochondrial respiratory chain complex I assembly;GO:0033108 mitochondrial respiratory chain complex assembly
FBgn0033571	Rpb5	GO:0009304 tRNA transcription;GO:0042797 tRNA transcription by RNA polymerase III;GO:0006360 transcription by RNA polymerase I
FBgn0265974	ttv	GO:0015014 heparan sulfate proteoglycan biosynthetic process, polysaccharide chain biosynthetic process;GO:0006024 glycosaminoglycan biosynthetic process;GO:0006044 N-acetylglucosamine metabolic process
FBgn0033969	Pgm2b	GO:0005975 carbohydrate metabolic process;GO:0044238 primary metabolic process;GO:0071704 organic substance metabolic process
FBgn0259966	Sfp51E	GO:0032504 multicellular organism reproduction;GO:0000003 reproduction;GO:0032501 multicellular organismal process

FBgn0264377	CG43829	GO:0008150 biological_process
FBgn0034012	Hr51	GO:0048842 positive regulation of axon extension involved in axon guidance;GO:0050921 positive regulation of chemotaxis;GO:0048526 imaginal disc-derived wing expansion
FBgn0034053	Cyp4aa1	GO:0006697 ecdysone biosynthetic process;GO:0008205 ecdysone metabolic process;GO:1902653 secondary alcohol biosynthetic process
FBgn0050093	COX6AL	GO:0006123 mitochondrial electron transport, cytochrome c to oxygen;GO:0019646 aerobic electron transport chain;GO:0042775 mitochondrial ATP synthesis coupled electron transport
FBgn0263576	mir-4919	NA
FBgn0265045	Strn-Mlck	GO:0006468 protein phosphorylation;GO:0016310 phosphorylation;GO:0006796 phosphate-containing compound metabolic process
FBgn0263391	hts	GO:0030724 testicular fusome organization;GO:0008302 female germline ring canal formation, actin assembly;GO:0007301 female germline ring canal formation
FBgn0012051	CalpA	GO:0016540 protein autoprocessing;GO:0000768 syncytium formation by plasma membrane fusion;GO:0006949 syncytium formation
FBgn0003435	sm	GO:0008343 adult feeding behavior;GO:0007631 feeding behavior;GO:0030534 adult behavior
FBgn0053535	tRNA:Glu-TTC-1-3	GO:0006412 translation;GO:0043043 peptide biosynthetic process;GO:0043604 amide biosynthetic process
FBgn0028622	qsm	GO:0045475 locomotor rhythm;GO:0048512 circadian behavior;GO:0007622 rhythmic behavior
FBgn0003731	Egfr	GO:0008071 maternal determination of dorsal/ventral axis, ovarian follicular epithelium, soma encoded;GO:0035310 notum cell fate specification;GO:0043703 photoreceptor cell fate determination
FBgn0265938	lncRNA:CR44725	NA
FBgn0020307	dve	GO:0015677 copper ion import;GO:0006825 copper ion transport;GO:0016348 imaginal disc-derived leg joint morphogenesis
FBgn0041236	Gr59d	GO:0050909 sensory perception of taste;GO:0007606 sensory perception of chemical stimulus;GO:0007600 sensory perception
FBgn0015903	apt	GO:0007319 negative regulation of oskar mRNA translation;GO:0035289 posterior head segmentation;GO:0046011 regulation of oskar mRNA translation
FBgn0034883	Eglp2	GO:0003014 renal system process;GO:0003008 system process;GO:0055085 transmembrane transport

FBgn0034885	Eglp4	GO:0003014 renal system process;GO:0003008 system process;GO:0055085 transmembrane transport
FBgn0024277	trio	GO:0007412 axon target recognition;GO:0035025 positive regulation of Rho protein signal transduction;GO:0035023 regulation of Rho protein signal transduction
FBgn0267487	Ptp61F	GO:0030947 regulation of vascular endothelial growth factor receptor signaling pathway;GO:0030948 negative regulation of vascular endothelial growth factor receptor signaling pathway;GO:0014067 negative regulation of phosphatidylinositol 3-kinase signaling
FBgn0260657	CG42540	NA
FBgn0035574	RhoGEF64C	GO:0035025 positive regulation of Rho protein signal transduction;GO:0035023 regulation of Rho protein signal transduction;GO:0035277 spiracle morphogenesis, open tracheal system
FBgn0267645	lncRNA:CR45983	NA
FBgn0035710	SP1173	GO:0008150 biological_process
FBgn0052364	tut	GO:0098729 germline stem cell symmetric division;GO:0098730 male germline stem cell symmetric division;GO:0098724 symmetric stem cell division
FBgn0035857	CG8006	GO:0008150 biological_process
FBgn0267796	Tmc	GO:0019232 perception of rate of movement;GO:1905790 regulation of mechanosensory behavior;GO:1905792 positive regulation of mechanosensory behavior
FBgn0036031	CG6761	NA
FBgn0036032	CG16711	GO:0008150 biological_process
FBgn0085385	bma	GO:0006468 protein phosphorylation;GO:0016310 phosphorylation;GO:0006796 phosphate-containing compound metabolic process
FBgn0036062	CG6685	NA
FBgn0026404	Dronc	GO:0010940 positive regulation of necrotic cell death;GO:2001269 positive regulation of cysteine-type endopeptidase activity involved in apoptotic signaling pathway;GO:0010939 regulation of necrotic cell death
FBgn0262592	CG43127	NA
FBgn0085267	CG34238	GO:0008150 biological_process

FBgn0036089	CG14151	GO:0008150 biological_process
FBgn0260644	CG42536	GO:0008150 biological_process
FBgn0036363	CG10140	NA
FBgn0036364	CG14109	NA
FBgn0036365	cmb	GO:0035317 imaginal disc-derived wing hair organization;GO:0035315 hair cell differentiation;GO:0035316 non-sensory hair organization
FBgn0087007	bbg	GO:0007298 border follicle cell migration;GO:0007297 ovarian follicle cell migration;GO:0010631 epithelial cell migration
FBgn0036544	sff	GO:0042149 cellular response to glucose starvation;GO:0006487 protein N-linked glycosylation;GO:0030010 establishment of cell polarity
FBgn0036556	hzg	GO:0040019 positive regulation of embryonic development;GO:0045995 regulation of embryonic development;GO:0006470 protein dephosphorylation
FBgn0036846	MESR6	NA
FBgn0036847	CNPYb	GO:0061077 chaperone-mediated protein folding;GO:0006457 protein folding;GO:0009987 cellular process
FBgn0036848	Naxd	GO:0110051 metabolite repair;GO:0044237 cellular metabolic process;GO:0008152 metabolic process
FBgn0036849	CG14079	GO:0007283 spermatogenesis;GO:0048232 male gamete generation;GO:0003006 developmental process involved in reproduction
FBgn0264785	Hph	GO:0006611 protein export from nucleus;GO:0018401 peptidyl-proline hydroxylation to 4-hydroxy-L-proline;GO:0007430 terminal branching, open tracheal system
FBgn0266393	CR45033	NA
FBgn0013576	mtd	GO:2000648 positive regulation of stem cell proliferation;GO:0002218 activation of innate immune response;GO:0002253 activation of immune response
FBgn0051481	pb	GO:0007381 specification of segmental identity, labial segment;GO:0007382 specification of segmental identity, maxillary segment;GO:0035213 clypeo-labral disc development
FBgn0085413	CG34384	GO:0007205 protein kinase C-activating G protein-coupled receptor signaling pathway;GO:0046834 lipid phosphorylation;GO:0046339 diacylglycerol metabolic process

FBgn0037525	CG17816	GO:0010826 negative regulation of centrosome duplication;GO:0046600 negative regulation of centriole replication;GO:0046606 negative regulation of centrosome cycle
FBgn0024909	Taf7	GO:0051123 RNA polymerase II preinitiation complex assembly;GO:0070897 transcription preinitiation complex assembly;GO:0006367 transcription initiation from RNA polymerase II promoter
FBgn0004901	Prat	GO:0009113 purine nucleobase biosynthetic process;GO:0040016 embryonic cleavage;GO:0006144 purine nucleobase metabolic process
FBgn0014930	CG2846	GO:0006771 riboflavin metabolic process;GO:0009231 riboflavin biosynthetic process;GO:0009398 FMN biosynthetic process
FBgn0014931	CG2678	GO:0006357 regulation of transcription by RNA polymerase II;GO:0006366 transcription by RNA polymerase II;GO:0006355 regulation of transcription, DNA-templated
FBgn0046874	Pif1B	GO:0008150 biological_process
FBgn0261015	Pif1A	NA
FBgn0053191	CG33191	GO:0008150 biological_process
FBgn0053189	CG33189	GO:0008150 biological_process
FBgn0267376	SelR	GO:0030091 protein repair;GO:0030240 skeletal muscle thin filament assembly;GO:0014866 skeletal myofibril assembly
FBgn0259227	CG42327	GO:0006470 protein dephosphorylation;GO:0016311 dephosphorylation;GO:0006796 phosphate-containing compound metabolic process
FBgn0037963	Cad87A	GO:0044331 cell-cell adhesion mediated by cadherin;GO:0016339 calcium-dependent cell-cell adhesion via plasma membrane cell adhesion molecules;GO:0007156 homophilic cell adhesion via plasma membrane adhesion molecules
FBgn0003319	Sb	GO:0051017 actin filament bundle assembly;GO:0061572 actin filament bundle organization;GO:0007015 actin filament organization
FBgn0040071	tara	GO:0048096 chromatin-mediated maintenance of transcription;GO:0045815 positive regulation of gene expression, epigenetic;GO:0006368 transcription elongation from RNA polymerase II promoter
FBgn0259244	CG42342	GO:0030198 extracellular matrix organization;GO:0043062 extracellular structure organization;GO:0045229 external encapsulating structure organization
FBgn0038488	m-cup	GO:0016567 protein ubiquitination;GO:0032446 protein modification by small protein conjugation;GO:0070647 protein modification by small protein conjugation or removal
FBgn0264291	Det	GO:0007111 meiosis II cytokinesis;GO:0000915 actomyosin contractile ring assembly;GO:0000912 assembly of actomyosin apparatus involved in cytokinesis

FBgn0038490	CG5285	GO:0006284 base-excision repair;GO:0006281 DNA repair;GO:0006974 cellular response to DNA damage stimulus
FBgn0038491	CG5292	GO:0002100 tRNA wobble adenosine to inosine editing;GO:0006382 adenosine to inosine editing;GO:0016553 base conversion or substitution editing
FBgn0038492	Mur89F	NA
FBgn0039016	Dcr-1	GO:0035087 siRNA loading onto RISC involved in RNA interference;GO:0031054 pre-miRNA processing;GO:0030422 production of siRNA involved in RNA interference
FBgn0011725	twin	GO:0048132 female germ-line stem cell asymmetric division;GO:0000289 nuclear-transcribed mRNA poly(A) tail shortening;GO:0098728 germline stem cell asymmetric division
FBgn0026257	cav	GO:0016233 telomere capping;GO:0000723 telomere maintenance;GO:0032200 telomere organization
FBgn0039169	Spps	GO:0060968 regulation of gene silencing;GO:0016458 gene silencing;GO:0010629 negative regulation of gene expression
FBgn0086365	Orct2	GO:0015695 organic cation transport;GO:0040018 positive regulation of multicellular organism growth;GO:0040014 regulation of multicellular organism growth
FBgn0019952	Orct	GO:0006915 apoptotic process;GO:0012501 programmed cell death;GO:0008219 cell death
FBgn0053341	CG33341	GO:0008150 biological_process
FBgn0039194	CG13614	NA
FBgn0039195	CG17782	NA
FBgn0039196	CG17781	NA
FBgn0267216	lncRNA:CR45656	NA
FBgn0267217	lncRNA:CR45657	NA
FBgn0040602	CG14545	NA
FBgn0039379	CG5886	NA

FBgn0039818	CG11318	GO:0007189 adenylate cyclase-activating G protein-coupled receptor signaling pathway;GO:0007188 adenylate cyclase-modulating G protein-coupled receptor signaling pathway;GO:0007186 G protein-coupled receptor signaling pathway
FBgn0040372	G9a	GO:0036123 histone H3-K9 dimethylation;GO:1900109 regulation of histone H3-K9 dimethylation;GO:1900111 positive regulation of histone H3-K9 dimethylation
FBgn0261930	vnd	GO:0007400 neuroblast fate determination;GO:0014019 neuroblast development;GO:0014017 neuroblast fate commitment
FBgn0025633	CG13366	GO:0048734 proboscis morphogenesis;GO:0048802 notum morphogenesis;GO:0035213 clypeo-labral disc development
FBgn0264449	CG43867	GO:0008150 biological_process
FBgn0264562	Hr4	GO:0033144 negative regulation of intracellular steroid hormone receptor signaling pathway;GO:0120143 negative regulation of ecdysone receptor-mediated signaling pathway;GO:1901420 negative regulation of response to alcohol
FBgn0003079	Raf	GO:0046534 positive regulation of photoreceptor cell differentiation;GO:0016242 negative regulation of macroautophagy;GO:0035309 wing and notum subfield formation
FBgn0054052	lncRNA:CR34052	GO:0008150 biological_process
FBgn0003371	sgg	GO:0043506 regulation of JUN kinase activity;GO:0043508 negative regulation of JUN kinase activity;GO:0070884 regulation of calcineurin-NFAT signaling cascade
FBgn0025388	CG12179	GO:0010825 positive regulation of centrosome duplication;GO:0046601 positive regulation of centriole replication;GO:0046599 regulation of centriole replication
FBgn0025739	pon	GO:0035050 embryonic heart tube development;GO:0055059 asymmetric neuroblast division;GO:0055057 neuroblast division
FBgn0083940	RhoU	GO:0032488 Cdc42 protein signal transduction;GO:0007266 Rho protein signal transduction;GO:0007265 Ras protein signal transduction
FBgn0266199	asRNA:CR44894	NA
FBgn0086045	snoRNA:Me18S-A627	NA
FBgn0003321	sbr	GO:0032241 positive regulation of nucleobase-containing compound transport;GO:0046833 positive regulation of RNA export from nucleus;GO:0046831 regulation of RNA export from nucleus
FBgn0030237	CG15209	NA

FBgn0052669	CG32669	GO:0006814 sodium ion transport;GO:0030001 metal ion transport;GO:0006812 cation transport
FBgn0004370	Ptp10D	GO:0040037 negative regulation of fibroblast growth factor receptor signaling pathway;GO:0030947 regulation of vascular endothelial growth factor receptor signaling pathway;GO:0030948 negative regulation of vascular endothelial growth factor receptor signaling pathway
FBgn0030331	CG15221	GO:0055085 transmembrane transport;GO:0006810 transport;GO:0051234 establishment of localization
FBgn0265597	rad	GO:0007615 anesthesia-resistant memory;GO:0008016 regulation of heart contraction;GO:1903522 regulation of blood circulation
FBgn0027621	Pfrx	GO:0006003 fructose 2,6-bisphosphate metabolic process;GO:0006000 fructose metabolic process;GO:0046835 carbohydrate phosphorylation
FBgn0031023	CG14200	NA
FBgn0053517	Dop2R	GO:0007212 dopamine receptor signaling pathway;GO:0071875 adrenergic receptor signaling pathway;GO:0071880 adenylate cyclase-activating adrenergic receptor signaling pathway
FBgn0031183	CG14621	NA
FBgn0031184	CG14615	NA
FBgn0014395	tilB	GO:0010378 temperature compensation of the circadian clock;GO:0036158 outer dynein arm assembly;GO:0045433 male courtship behavior, veined wing generated song production
FBgn0004611	Plc21C	GO:0043153 entrainment of circadian clock by photoperiod;GO:0009648 photoperiodism;GO:0007629 flight behavior
FBgn0003278	RpI135	GO:0006360 transcription by RNA polymerase I;GO:0006351 transcription, DNA-templated;GO:0097659 nucleic acid-templated transcription
FBgn0264855	AP-2alpha	GO:0038009 regulation of signal transduction by receptor internalization;GO:0038010 positive regulation of signal transduction by receptor internalization;GO:1990386 mitotic cleavage furrow ingression
FBgn0263657	lncRNA:CR43648	NA
FBgn0263658	lncRNA:CR43649	NA
FBgn0031275	GABA-B-R3	GO:0007214 gamma-aminobutyric acid signaling pathway;GO:0030431 sleep;GO:0007186 G protein-coupled receptor signaling pathway
FBgn0041097	robo3	GO:0048036 central complex development;GO:0016201 synaptic target inhibition;GO:0050919 negative chemotaxis

FBgn0000579	Eno	GO:0032889 regulation of vacuole fusion, non-autophagic;GO:0042144 vacuole fusion, non-autophagic;GO:0097576 vacuole fusion
FBgn0260648	Rrp40	GO:0071031 nuclear mRNA surveillance of mRNA 3'-end processing;GO:0071049 nuclear retention of pre-mRNA with aberrant 3'-ends at the site of transcription;GO:0071034 CUT catabolic process
FBgn0031360	CG31937	NA
FBgn0031361	CG17652	NA
FBgn0264494	CG17646	GO:0006641 triglyceride metabolic process;GO:0006638 neutral lipid metabolic process;GO:0006639 acylglycerol metabolic process
FBgn0053543	CG33543	NA
FBgn0040718	CG15353	NA
FBgn0267276	asRNA:CR45712	NA
FBgn0040717	Nplp4	GO:0007218 neuropeptide signaling pathway;GO:0007186 G protein-coupled receptor signaling pathway;GO:0007165 signal transduction
FBgn0051943	tRNA:Gln-CTG-2-2	GO:0006412 translation;GO:0043043 peptide biosynthetic process;GO:0043604 amide biosynthetic process
FBgn0051944	tRNA:Gln-CTG-2-3	GO:0006412 translation;GO:0043043 peptide biosynthetic process;GO:0043604 amide biosynthetic process
FBgn0026396	Or22c	GO:0050911 detection of chemical stimulus involved in sensory perception of smell;GO:0050907 detection of chemical stimulus involved in sensory perception;GO:0007608 sensory perception of smell
FBgn0053516	dpr3	GO:0002115 store-operated calcium entry;GO:0006816 calcium ion transport;GO:0030001 metal ion transport
FBgn0000097	aop	GO:0035155 negative regulation of terminal cell fate specification, open tracheal system;GO:0035157 negative regulation of fusion cell fate specification;GO:0060233 oenocyte delamination
FBgn0031398	daed	GO:0008150 biological_process
FBgn0040715	CG15386	GO:0017062 respiratory chain complex III assembly;GO:0034551 mitochondrial respiratory chain complex III assembly;GO:0017004 cytochrome complex assembly
FBgn0259717	CG42371	NA

FBgn0031399	mio	GO:0040020 regulation of meiotic nuclear division;GO:0034198 cellular response to amino acid starvation;GO:1990928 response to amino acid starvation
FBgn0031401	papi	GO:0070895 negative regulation of transposon integration;GO:0015074 DNA integration;GO:0070893 transposon integration
FBgn0031548	CG8852	GO:0008150 biological_process
FBgn0031549	Spindly	GO:0030071 regulation of mitotic metaphase/anaphase transition;GO:1902099 regulation of metaphase/anaphase transition of cell cycle;GO:0007091 metaphase/anaphase transition of mitotic cell cycle
FBgn0051774	fred	GO:0035018 adult chitin-based cuticle pattern formation;GO:0008365 adult chitin-based cuticle development;GO:0090175 regulation of establishment of planar polarity
FBgn0051773	CG31773	GO:0046901 tetrahydrofolylpolyglutamate biosynthetic process;GO:0046900 tetrahydrofolylpolyglutamate metabolic process;GO:0009396 folic acid-containing compound biosynthetic process
FBgn0267441	lncRNA:CR45791	NA
FBgn0264866	lncRNA:CR44057	NA
FBgn0051961	TBCC	GO:0007023 post-chaperonin tubulin folding pathway;GO:0007021 tubulin complex assembly;GO:0006457 protein folding
FBgn0001942	eIF4A	GO:0048132 female germ-line stem cell asymmetric division;GO:0002183 cytoplasmic translational initiation;GO:0098728 germline stem cell asymmetric division
FBgn0001941	ifc	GO:0007053 spindle assembly involved in male meiosis;GO:0046513 ceramide biosynthetic process;GO:0006672 ceramide metabolic process
FBgn0031763	CG13996	GO:0008150 biological_process
FBgn0031764	CG9107	GO:0000028 ribosomal small subunit assembly;GO:0042255 ribosome assembly;GO:0042274 ribosomal small subunit biogenesis
FBgn0031765	CG9109	NA
FBgn0026755	Ugt37B1	NA
FBgn0031850	Tsp	GO:0033627 cell adhesion mediated by integrin;GO:0016203 muscle attachment;GO:0060538 skeletal muscle organ development
FBgn0031879	uif	GO:0045167 asymmetric protein localization involved in cell fate determination;GO:0016360 sensory organ precursor cell fate determination;GO:0060582 cell fate determination involved in pattern specification

FBgn0032153	CG4537	GO:0031122 cytoplasmic microtubule organization;GO:0097435 supramolecular fiber organization;GO:0000226 microtubule cytoskeleton organization
FBgn0032154	mtDNA-helicase	GO:0000002 mitochondrial genome maintenance;GO:0006264 mitochondrial DNA replication;GO:0006261 DNA-dependent DNA replication
FBgn0063449	Uhg2	NA
FBgn0063391	snoRNA:U14:30Eb	NA
FBgn0063392	snoRNA:U14:30Ea	NA
FBgn0063389	snoRNA:Me18S-G1620	NA
FBgn0025881	snoRNA:Me18S-A1576	NA
FBgn0010520	Bka	NA
FBgn0032313	CG14070	NA
FBgn0264442	ab	GO:0016198 axon choice point recognition;GO:0016203 muscle attachment;GO:0060538 skeletal muscle organ development
FBgn0052831	CG33695	NA
FBgn0265071	asRNA:CR44182	NA
FBgn0032358	Ppt2	GO:0098734 macromolecule depalmitoylation;GO:0098732 macromolecule deacylation;GO:0043412 macromolecule modification
FBgn0020270	mre11	GO:0097551 mitochondrial double-strand break repair;GO:0097552 mitochondrial double-strand break repair via homologous recombination;GO:0042138 meiotic DNA double-strand break formation
FBgn0028425	JhI-21	GO:0043201 response to leucine;GO:0071233 cellular response to leucine;GO:0015803 branched-chain amino acid transport
FBgn0032404	RpL7-like	GO:0000463 maturation of LSU-rRNA from tricistronic rRNA transcript (SSU-rRNA, 5.8S rRNA, LSU-rRNA);GO:0000470 maturation of LSU-rRNA;GO:0042273 ribosomal large subunit biogenesis

FBgn0085193	CG34164	GO:0008150 biological_process
FBgn0027505	Rab3-GAP	GO:0060025 regulation of synaptic activity;GO:0043087 regulation of GTPase activity;GO:0016236 macroautophagy
FBgn0021796	Tor	GO:2001023 regulation of response to drug;GO:0090070 positive regulation of ribosome biogenesis;GO:0031670 cellular response to nutrient
FBgn0032467	CG9934	GO:0030433 ubiquitin-dependent ERAD pathway;GO:0036503 ERAD pathway;GO:0034976 response to endoplasmic reticulum stress
FBgn0267017	lncRNA:CR45461	NA
FBgn0032470	Ttc30	GO:0018095 protein polyglutamylation;GO:0018200 peptidyl-glutamic acid modification;GO:0042073 intraciliary transport
FBgn0028540	CG9008	GO:0051607 defense response to virus;GO:0140546 defense response to symbiont;GO:0009615 response to virus
FBgn0051845	asRNA:CR31845	GO:0008150 biological_process
FBgn0024846	p38b	GO:0071243 cellular response to arsenic-containing substance;GO:0071276 cellular response to cadmium ion;GO:0038001 paracrine signaling
FBgn0259735	mtgo	GO:0008150 biological_process
FBgn0000183	BicD	GO:2000302 positive regulation of synaptic vesicle exocytosis;GO:1903307 positive regulation of regulated secretory pathway;GO:0045921 positive regulation of exocytosis
FBgn0032640	Sgt	GO:1903334 positive regulation of protein folding;GO:1903646 positive regulation of chaperone-mediated protein folding;GO:1903332 regulation of protein folding
FBgn0015609	CadN	GO:0050774 negative regulation of dendrite morphogenesis;GO:0045463 R8 cell development;GO:0048841 regulation of axon extension involved in axon guidance
FBgn0023096	btv	GO:0035721 intraciliary retrograde transport;GO:0031223 auditory behavior;GO:0045433 male courtship behavior, veined wing generated song production
FBgn0032656	CG5674	NA
FBgn0032694	MESR3	GO:0006357 regulation of transcription by RNA polymerase II;GO:0006366 transcription by RNA polymerase II;GO:0006355 regulation of transcription, DNA-templated
FBgn0002022	Catsup	GO:0042069 regulation of catecholamine metabolic process;GO:0033238 regulation of cellular amine metabolic process;GO:0006882 cellular zinc ion homeostasis

FBgn0032744	Ttc19	GO:0007634 optokinetic behavior;GO:0071632 optomotor response;GO:0017062 respiratory chain complex III assembly
FBgn0263198	Acn	GO:0000335 negative regulation of transposition, DNA-mediated;GO:0000337 regulation of transposition, DNA-mediated;GO:0006313 transposition, DNA-mediated
FBgn0041789	Pax	GO:0060142 regulation of syncytium formation by plasma membrane fusion;GO:1901739 regulation of myoblast fusion;GO:0007160 cell-matrix adhesion
FBgn0000464	Lar	GO:1903386 negative regulation of homophilic cell adhesion;GO:1903385 regulation of homophilic cell adhesion;GO:0061484 hematopoietic stem cell homeostasis
FBgn0267964	CG46244	NA
FBgn0005590	scw	GO:0007502 digestive tract mesoderm development;GO:0010862 positive regulation of pathway-restricted SMAD protein phosphorylation;GO:0007378 amnioserosa formation
FBgn0263873	sick	GO:0050829 defense response to Gram-negative bacterium;GO:0007015 actin filament organization;GO:0042742 defense response to bacterium
FBgn0032857	CG10947	GO:0006417 regulation of translation;GO:0034248 regulation of cellular amide metabolic process;GO:0010608 posttranscriptional regulation of gene expression
FBgn0032901	sky	GO:1903422 negative regulation of synaptic vesicle recycling;GO:0046929 negative regulation of neurotransmitter secretion;GO:0051589 negative regulation of neurotransmitter transport
FBgn0263996	CG43739	GO:0008150 biological_process
FBgn0040297	Nhe2	GO:0045852 pH elevation;GO:0051454 intracellular pH elevation;GO:0098719 sodium ion import across plasma membrane
FBgn0284223	CG46307	NA
FBgn0023090	dtr	GO:0044458 motile cilium assembly;GO:0060271 cilium assembly;GO:0044782 cilium organization
FBgn0028979	tio	GO:0061330 Malpighian tubule stellate cell differentiation;GO:0048730 epidermis morphogenesis;GO:0007380 specification of segmental identity, head
FBgn0085414	dpr12	GO:0008039 synaptic target recognition;GO:0008038 neuron recognition;GO:0008037 cell recognition
FBgn0263109	CG43366	GO:0008150 biological_process
FBgn0033048	CG7881	GO:0006820 anion transport;GO:0006811 ion transport;GO:0055085 transmembrane transport

FBgn0033054	CG14591	NA
FBgn0086655	jing	GO:0007382 specification of segmental identity, maxillary segment;GO:0007418 ventral midline development;GO:0007380 specification of segmental identity, head
FBgn0033076	CG15233	NA
FBgn0261397	didum	GO:0045856 positive regulation of pole plasm oskar mRNA localization;GO:1904582 positive regulation of intracellular mRNA localization;GO:0030050 vesicle transport along actin filament
FBgn0033184	mEFTu2	GO:0070125 mitochondrial translational elongation;GO:0006414 translational elongation;GO:0032543 mitochondrial translation
FBgn0025185	az2	GO:0006357 regulation of transcription by RNA polymerase II;GO:0006366 transcription by RNA polymerase II;GO:0006355 regulation of transcription, DNA-templated
FBgn0033203	CG2070	GO:0042574 retinal metabolic process;GO:0120254 olefinic compound metabolic process;GO:0001523 retinoid metabolic process
FBgn0033204	CG2065	GO:0042574 retinal metabolic process;GO:0120254 olefinic compound metabolic process;GO:0001523 retinoid metabolic process
FBgn0033205	CG2064	GO:0042574 retinal metabolic process;GO:0120254 olefinic compound metabolic process;GO:0001523 retinoid metabolic process
FBgn0033206	DCTN4-p62	NA
FBgn0263593	Lpin	GO:2000001 regulation of DNA damage checkpoint;GO:0019217 regulation of fatty acid metabolic process;GO:1901976 regulation of cell cycle checkpoint
FBgn0010504	kermit	GO:0008045 motor neuron axon guidance;GO:0001736 establishment of planar polarity;GO:0007164 establishment of tissue polarity
FBgn0264877	lncRNA:CR44068	NA
FBgn0050374	CR30374	NA
FBgn0050297	tRNA:Ile-AAT-1-2	GO:0006412 translation;GO:0043043 peptide biosynthetic process;GO:0043604 amide biosynthetic process
FBgn0033271	CG8708	GO:0016267 O-glycan processing, core 1;GO:0016266 O-glycan processing;GO:0006493 protein O-linked glycosylation
FBgn0033322	CG8584	GO:0006470 protein dephosphorylation;GO:0016311 dephosphorylation;GO:0006796 phosphate-containing compound metabolic process

FBgn0033323	CG12376	GO:0006874 cellular calcium ion homeostasis;GO:0055074 calcium ion homeostasis;GO:0072503 cellular divalent inorganic cation homeostasis
FBgn0260972	alc	GO:0035090 maintenance of apical/basal cell polarity;GO:0030011 maintenance of cell polarity;GO:0070050 neuron cellular homeostasis
FBgn0028955	CG8788	NA
FBgn0265313	CG44286	GO:0008150 biological_process
FBgn0086656	shrb	GO:0032511 late endosome to vacuole transport via multivesicular body sorting pathway;GO:0043162 ubiquitin-dependent protein catabolic process via the multivesicular body sorting pathway;GO:0048132 female germ-line stem cell asymmetric division
FBgn0266819	asRNA:CR45281	NA
FBgn0033422	Or45b	GO:0050911 detection of chemical stimulus involved in sensory perception of smell;GO:0050907 detection of chemical stimulus involved in sensory perception;GO:0007608 sensory perception of smell
FBgn0033423	Alp6	GO:0016311 dephosphorylation;GO:0006796 phosphate-containing compound metabolic process;GO:0006793 phosphorus metabolic process
FBgn0040765	luna	GO:0035185 preblastoderm mitotic cell cycle;GO:0033301 cell cycle comprising mitosis without cytokinesis;GO:0045448 mitotic cell cycle, embryonic
FBgn0033616	CG7745	GO:0006357 regulation of transcription by RNA polymerase II;GO:0006366 transcription by RNA polymerase II;GO:0006355 regulation of transcription, DNA-templated
FBgn0016047	nompA	GO:0000184 nuclear-transcribed mRNA catabolic process, nonsense-mediated decay;GO:0007605 sensory perception of sound;GO:0000956 nuclear-transcribed mRNA catabolic process
FBgn0262574	CG43114	GO:0008150 biological_process
FBgn0033656	S2P	GO:0036003 positive regulation of transcription from RNA polymerase II promoter in response to stress;GO:1990440 positive regulation of transcription from RNA polymerase II promoter in response to endoplasmic reticulum stress;GO:0035103 sterol regulatory element binding protein cleavage
FBgn0262819	CG43190	GO:0008150 biological_process
FBgn0265363	asRNA:CR44304	NA
FBgn0033657	Sln	GO:0015718 monocarboxylic acid transport;GO:0046942 carboxylic acid transport;GO:0015711 organic anion transport

FBgn0262889	CG43244	GO:0008150 biological_process
FBgn0265374	lncRNA:CR44315	NA
FBgn0033785	Sans	GO:0007605 sensory perception of sound;GO:0050954 sensory perception of mechanical stimulus;GO:0007600 sensory perception
FBgn0050487	CG30487	GO:0070301 cellular response to hydrogen peroxide;GO:0042542 response to hydrogen peroxide;GO:0034614 cellular response to reactive oxygen species
FBgn0004512	Mdr49	GO:0006855 drug transmembrane transport;GO:0015893 drug transport;GO:0042908 xenobiotic transport
FBgn0013765	cnn	GO:0030997 regulation of centriole-centriole cohesion;GO:0010457 centriole-centriole cohesion;GO:0040016 embryonic cleavage
FBgn0050062	CG30062	GO:0050829 defense response to Gram-negative bacterium;GO:0042742 defense response to bacterium;GO:0009617 response to bacterium
FBgn0033853	CG6145	GO:0006741 NADP biosynthetic process;GO:0019359 nicotinamide nucleotide biosynthetic process;GO:0019363 pyridine nucleotide biosynthetic process
FBgn0053156	CG33156	GO:0006741 NADP biosynthetic process;GO:0019359 nicotinamide nucleotide biosynthetic process;GO:0019363 pyridine nucleotide biosynthetic process
FBgn0050484	CG30484	GO:0008150 biological_process
FBgn0050485	CG30485	GO:0008150 biological_process
FBgn0283553	mir-9379	NA
FBgn0033879	Echs1	GO:0006635 fatty acid beta-oxidation;GO:0019395 fatty acid oxidation;GO:0034440 lipid oxidation
FBgn0033982	Cyp317a1	NA
FBgn0027596	Kank	GO:0030837 negative regulation of actin filament polymerization;GO:0032272 negative regulation of protein polymerization;GO:0031333 negative regulation of protein-containing complex assembly
FBgn0034046	tun	GO:0006464 cellular protein modification process;GO:0036211 protein modification process;GO:0043412 macromolecule modification
FBgn0034070	SP2353	GO:0045824 negative regulation of innate immune response;GO:0046716 muscle cell cellular homeostasis;GO:0042052 rhabdomere development

FBgn0034071	CG8405	GO:1904294 positive regulation of ERAD pathway;GO:1905898 positive regulation of response to endoplasmic reticulum stress;GO:1904292 regulation of ERAD pathway
FBgn0034072	Dg	GO:0006110 regulation of glycolytic process;GO:1903798 regulation of production of miRNAs involved in gene silencing by miRNA;GO:0070920 regulation of production of small RNA involved in gene silencing by RNA
FBgn0034085	Ptp52F	GO:0035335 peptidyl-tyrosine dephosphorylation;GO:0035096 larval midgut cell programmed cell death;GO:0035069 larval midgut histolysis
FBgn0264273	Sema2b	GO:0071678 olfactory bulb axon guidance;GO:0016200 synaptic target attraction;GO:0050918 positive chemotaxis
FBgn0034366	Atg7	GO:0006501 C-terminal protein lipidation;GO:0044805 late nucleophagy;GO:1903599 positive regulation of autophagy of mitochondrion
FBgn0266671	Sec6	GO:0051601 exocyst localization;GO:0045313 rhabdomere membrane biogenesis;GO:0072697 protein localization to cell cortex
FBgn0000566	Eip55E	GO:0009092 homoserine metabolic process;GO:0019346 transsulfuration;GO:0019344 cysteine biosynthetic process
FBgn0034379	CG15073	GO:0045944 positive regulation of transcription by RNA polymerase II;GO:0045893 positive regulation of transcription, DNA-templated;GO:1902680 positive regulation of RNA biosynthetic process
FBgn0034380	Vps51	GO:0007041 lysosomal transport;GO:0042147 retrograde transport, endosome to Golgi;GO:0032456 endocytic recycling
FBgn0062961	lncRNA:CR33942	NA
FBgn0263395	hppy	GO:0040009 regulation of growth rate;GO:1904263 positive regulation of TORC1 signaling;GO:0035332 positive regulation of hippo signaling
FBgn0034425	CG11906	GO:0006357 regulation of transcription by RNA polymerase II;GO:0006366 transcription by RNA polymerase II;GO:0006355 regulation of transcription, DNA-templated
FBgn0034426	AANATL5	NA
FBgn0034427	CG10474	GO:0006517 protein deglycosylation;GO:0009100 glycoprotein metabolic process;GO:1901135 carbohydrate derivative metabolic process
FBgn0034428	AANATL6	NA
FBgn0265838	lncRNA:CR44627	NA
FBgn0004364	18w	NA

FBgn0086604	side-VIII	NA
FBgn0034552	CG17999	GO:0046949 fatty-acyl-CoA biosynthetic process;GO:1901570 fatty acid derivative biosynthetic process;GO:0035384 thioester biosynthetic process
FBgn0010470	Fkbp14	GO:0046716 muscle cell cellular homeostasis;GO:0045747 positive regulation of Notch signaling pathway;GO:0008593 regulation of Notch signaling pathway
FBgn0034631	TAF1C-like	GO:0042790 nucleolar large rRNA transcription by RNA polymerase I;GO:0009303 rRNA transcription;GO:0006360 transcription by RNA polymerase I
FBgn0043070	MESK2	GO:0007165 signal transduction;GO:0023052 signaling;GO:0007154 cell communication
FBgn0067102	GlcT	GO:0006679 glucosylceramide biosynthetic process;GO:0046476 glycosylceramide biosynthetic process;GO:0006678 glucosylceramide metabolic process
FBgn0034691	Synj	GO:0044090 positive regulation of vacuole organization;GO:2000786 positive regulation of autophagosome assembly;GO:2000785 regulation of autophagosome assembly
FBgn0034750	CG3732	NA
FBgn0261596	RpS24	GO:0002181 cytoplasmic translation;GO:0006412 translation;GO:0043043 peptide biosynthetic process
FBgn0040091	Ugt317A1	NA
FBgn0034753	CG2852	GO:0000413 protein peptidyl-prolyl isomerization;GO:0018208 peptidyl-proline modification;GO:0006457 protein folding
FBgn0004795	retn	GO:0045924 regulation of female receptivity;GO:0060180 female mating behavior;GO:0008049 male courtship behavior
FBgn0004870	bab1	GO:0048088 regulation of male pigmentation;GO:0048092 negative regulation of male pigmentation;GO:0048094 male pigmentation
FBgn0035186	CG13912	NA
FBgn0035187	Trh	GO:0042427 serotonin biosynthetic process;GO:0042428 serotonin metabolic process;GO:1901160 primary amino compound metabolic process
FBgn0005640	Eip63E	GO:0000083 regulation of transcription involved in G1/S transition of mitotic cell cycle;GO:0000082 G1/S transition of mitotic cell cycle;GO:0044843 cell cycle G1/S phase transition
FBgn0262870	axo	GO:0019226 transmission of nerve impulse;GO:0035637 multicellular organismal signaling;GO:0050877 nervous system process

FBgn0052238	TTLL1B	GO:0018095 protein polyglutamylation;GO:0018094 protein polyglycylation;GO:0018200 peptidyl-glutamic acid modification
FBgn0052423	shep	GO:0008050 female courtship behavior;GO:0009629 response to gravity;GO:0042332 gravitaxis
FBgn0085447	sif	GO:0050772 positive regulation of axonogenesis;GO:0007601 visual perception;GO:0051491 positive regulation of filopodium assembly
FBgn0284236	CG46320	NA
FBgn0261536	CG42660	GO:0008150 biological_process
FBgn0261537	CG42661	GO:0008150 biological_process
FBgn0035786	Tsp66A	GO:0008150 biological_process
FBgn0035787	CG8543	NA
FBgn0016694	Pdp1	GO:0019216 regulation of lipid metabolic process;GO:0007623 circadian rhythm;GO:0048511 rhythmic process
FBgn0262719	CG43163	GO:0008150 biological_process
FBgn0035901	Pus7	GO:0001522 pseudouridine synthesis;GO:0009451 RNA modification;GO:0043412 macromolecule modification
FBgn0035903	CG6765	GO:0006357 regulation of transcription by RNA polymerase II;GO:0006366 transcription by RNA polymerase II;GO:0006355 regulation of transcription, DNA-templated
FBgn0035902	CG6683	GO:0006357 regulation of transcription by RNA polymerase II;GO:0006366 transcription by RNA polymerase II;GO:0006355 regulation of transcription, DNA-templated
FBgn0015321	Ubc4	GO:0045880 positive regulation of smoothened signaling pathway;GO:0008589 regulation of smoothened signaling pathway;GO:0007224 smoothened signaling pathway
FBgn0052062	Rbfox1	GO:0030706 germarium-derived oocyte differentiation;GO:0062197 cellular response to chemical stress;GO:0007474 imaginal disc-derived wing vein specification
FBgn0259481	Mob2	GO:0045886 negative regulation of synaptic assembly at neuromuscular junction;GO:1904397 negative regulation of neuromuscular junction development;GO:0042052 rhabdomere development
FBgn0052085	CG32085	GO:0031146 SCF-dependent proteasomal ubiquitin-dependent protein catabolic process;GO:0043161 proteasome-mediated ubiquitin-dependent protein catabolic process;GO:0010498 proteasomal protein catabolic process

FBgn0015919	caup	GO:0045317 equator specification;GO:0042693 muscle cell fate commitment;GO:0048859 formation of anatomical boundary
FBgn0264001	bru3	GO:0006376 mRNA splice site selection;GO:0000245 spliceosomal complex assembly;GO:0017148 negative regulation of translation
FBgn0001085	fz	GO:0022606 establishment of proximal/distal cell polarity;GO:0035320 imaginal disc-derived wing hair site selection;GO:0035567 non-canonical Wnt signaling pathway
FBgn0036421	CG13481	GO:0036297 interstrand cross-link repair;GO:0006281 DNA repair;GO:0016567 protein ubiquitination
FBgn0036485	FucTA	GO:0036071 N-glycan fucosylation;GO:0036065 fucosylation;GO:0006486 protein glycosylation
FBgn0036486	Msh6	GO:0043570 maintenance of DNA repeat elements;GO:0006290 pyrimidine dimer repair;GO:0000710 meiotic mismatch repair
FBgn0005536	Mbs	GO:1904059 regulation of locomotor rhythm;GO:0045314 regulation of compound eye photoreceptor development;GO:0042478 regulation of eye photoreceptor cell development
FBgn0053258	CG33258	NA
FBgn0036563	CG13075	NA
FBgn0263602	Tasp1	GO:0016485 protein processing;GO:0050829 defense response to Gram-negative bacterium;GO:0051604 protein maturation
FBgn0000017	Abl	GO:0021785 branchiomotor neuron axon guidance;GO:0046827 positive regulation of protein export from nucleus;GO:0046825 regulation of protein export from nucleus
FBgn0266776	lncRNA:CR45241	NA
FBgn0040512	zetaCOP	GO:0006890 retrograde vesicle-mediated transport, Golgi to endoplasmic reticulum;GO:0006891 intra-Golgi vesicle-mediated transport;GO:0010883 regulation of lipid storage
FBgn0036652	CG13032	GO:1905515 non-motile cilium assembly;GO:0031122 cytoplasmic microtubule organization;GO:0060271 cilium assembly
FBgn0036659	CG9701	GO:0005975 carbohydrate metabolic process;GO:0044238 primary metabolic process;GO:0071704 organic substance metabolic process
FBgn0000567	Eip74EF	GO:0040034 regulation of development, heterochronic;GO:0035071 salivary gland cell autophagic cell death;GO:0048102 autophagic cell death
FBgn0000568	Eip75B	GO:0007553 regulation of ecdysteroid metabolic process;GO:0019218 regulation of steroid metabolic process;GO:0032350 regulation of hormone metabolic process

FBgn0086081	snoRNA:Me28S-A30	NA
FBgn0036773	CG13698	NA
FBgn0036871	CG14096	NA
FBgn0052214	CG32214	GO:0008150 biological_process
FBgn0036910	Cyp305a1	NA
FBgn0023094	cyc	GO:0003053 circadian regulation of heart rate;GO:0048148 behavioral response to cocaine;GO:0008062 eclosion rhythm
FBgn0036911	Fibp	NA
FBgn0267238	lncRNA:CR45678	NA
FBgn0026179	siz	GO:0032014 positive regulation of ARF protein signal transduction;GO:0032012 regulation of ARF protein signal transduction;GO:0032011 ARF protein signal transduction
FBgn0020294	ko	GO:0008045 motor neuron axon guidance;GO:0007411 axon guidance;GO:0097485 neuron projection guidance
FBgn0086913	Rab26	GO:0044409 entry into host;GO:0046718 viral entry into host cell;GO:0051701 biological process involved in interaction with host
FBgn0000560	eg	GO:0007417 central nervous system development;GO:0006357 regulation of transcription by RNA polymerase II;GO:0006366 transcription by RNA polymerase II
FBgn0264487	CG43895	GO:0008150 biological_process
FBgn0037166	CG11426	GO:0046839 phospholipid dephosphorylation;GO:0007602 phototransduction;GO:0009583 detection of light stimulus
FBgn0266931	lncRNA:CR45382	NA
FBgn0037167	CG11425	GO:0046839 phospholipid dephosphorylation;GO:0030258 lipid modification;GO:0006644 phospholipid metabolic process
FBgn0037218	aux	GO:0072318 clathrin coat disassembly;GO:0072319 vesicle uncoating;GO:0072583 clathrin-dependent endocytosis

FBgn0261436	DhpD	GO:0006147 guanine catabolic process;GO:0046098 guanine metabolic process;GO:0051067 dihydropteridine metabolic process
FBgn0265082	Cdep	GO:0016601 Rac protein signal transduction;GO:0007265 Ras protein signal transduction;GO:0007264 small GTPase mediated signal transduction
FBgn0037312	CG11999	GO:0051084 'de novo' posttranslational protein folding;GO:0051085 chaperone cofactor-dependent protein refolding;GO:0006458 'de novo' protein folding
FBgn0037313	CG1161	NA
FBgn0044823	Spec2	GO:0035023 regulation of Rho protein signal transduction;GO:0007266 Rho protein signal transduction;GO:0046578 regulation of Ras protein signal transduction
FBgn0037350	CG2911	GO:0017182 peptidyl-diphthamide metabolic process;GO:0017183 peptidyl-diphthamide biosynthetic process from peptidyl-histidine;GO:1900247 regulation of cytoplasmic translational elongation
FBgn0037351	RpL13A	GO:0017148 negative regulation of translation;GO:0034249 negative regulation of cellular amide metabolic process;GO:0002181 cytoplasmic translation
FBgn0260002	snoRNA:Me28S-U2134b	NA
FBgn0086051	snoRNA:Me28S-U2134a	NA
FBgn0051551	CG31551	NA
FBgn0261261	plx	GO:0033627 cell adhesion mediated by integrin;GO:0090630 activation of GTPase activity;GO:0043547 positive regulation of GTPase activity
FBgn0037421	CG15594	NA
FBgn0010355	Taf1	GO:0044154 histone H3-K14 acetylation;GO:0010390 histone monoubiquitination;GO:0016574 histone ubiquitination
FBgn0001112	Gld	GO:0008364 pupal chitin-based cuticle development;GO:0046693 sperm storage;GO:0035209 pupal development
FBgn0261929	5-HT2B	GO:0007210 serotonin receptor signaling pathway;GO:0071867 response to monoamine;GO:0071868 cellular response to monoamine stimulus
FBgn0024326	Mkk4	GO:0048082 regulation of adult chitin-containing cuticle pigmentation;GO:0048079 regulation of cuticle pigmentation;GO:0048085 adult chitin-containing cuticle pigmentation

FBgn0037602	SLIRP2	NA
FBgn0027503	CG11970	NA
FBgn0260243	E(var)3-9	GO:0070827 chromatin maintenance;GO:0043954 cellular component maintenance;GO:0045944 positive regulation of transcription by RNA polymerase II
FBgn0037648	CG11975	GO:0034497 protein localization to phagophore assembly site;GO:0044804 autophagy of nucleus;GO:0000422 autophagy of mitochondrion
FBgn0037772	Spn85F	NA
FBgn0037773	CG5359	GO:0007018 microtubule-based movement;GO:0007017 microtubule-based process;GO:0006928 movement of cell or subcellular component
FBgn0053208	Mical	GO:0060386 synapse assembly involved in innervation;GO:0030047 actin modification;GO:0030240 skeletal muscle thin filament assembly
FBgn0037989	ATP8B	GO:0045332 phospholipid translocation;GO:0034204 lipid translocation;GO:0097035 regulation of membrane lipid distribution
FBgn0020496	CtBP	GO:0016360 sensory organ precursor cell fate determination;GO:0060582 cell fate determination involved in pattern specification;GO:0008052 sensory organ boundary specification
FBgn0038147	CCHa2	GO:0007218 neuropeptide signaling pathway;GO:0007186 G protein-coupled receptor signaling pathway;GO:0007165 signal transduction
FBgn0040553	CG14374	NA
FBgn0038148	CG14377	GO:0008150 biological_process
FBgn0038149	GILT1	GO:1900426 positive regulation of defense response to bacterium;GO:1900424 regulation of defense response to bacterium;GO:0031349 positive regulation of defense response
FBgn0011582	Dop1R1	GO:0007191 adenylate cyclase-activating dopamine receptor signaling pathway;GO:0008542 visual learning;GO:0099509 regulation of presynaptic cytosolic calcium ion concentration
FBgn0038221	IFT54	GO:0042073 intraciliary transport;GO:0070507 regulation of microtubule cytoskeleton organization;GO:0032886 regulation of microtubule-based process
FBgn0003567	su(Hw)	GO:1905632 protein localization to euchromatin;GO:0071168 protein localization to chromatin;GO:0031936 negative regulation of chromatin silencing
FBgn0261859	CG42788	GO:0007165 signal transduction;GO:0023052 signaling;GO:0007154 cell communication

FBgn0038447	CG14892	GO:0006508 proteolysis;GO:0019538 protein metabolic process;GO:1901564 organonitrogen compound metabolic process
FBgn0027562	CG10345	GO:0001562 response to protozoan;GO:0042832 defense response to protozoan;GO:0098542 defense response to other organism
FBgn0264857	lncRNA:iab8	GO:0008150 biological_process
FBgn0020546	lncRNA:iab4	NA
FBgn0262290	mir-iab-8	NA
FBgn0262462	mir-iab-4	NA
FBgn0015008	Actn3	GO:0030036 actin cytoskeleton organization;GO:0030029 actin filament-based process;GO:0007010 cytoskeleton organization
FBgn0015011	AhcyL2	GO:0033353 S-adenosylmethionine cycle;GO:0046500 S-adenosylmethionine metabolic process;GO:0006790 sulfur compound metabolic process
FBgn0038465	Irc	GO:0098869 cellular oxidant detoxification;GO:0097237 cellular response to toxic substance;GO:1990748 cellular detoxification
FBgn0038466	CG8907	GO:0042048 olfactory behavior;GO:0007635 chemosensory behavior;GO:0007610 behavior
FBgn0002941	slou	GO:0007521 muscle cell fate determination;GO:0042693 muscle cell fate commitment;GO:0007517 muscle organ development
FBgn0038938	CG7084	GO:0055085 transmembrane transport;GO:0006810 transport;GO:0051234 establishment of localization
FBgn0267385	PyK	GO:0009744 response to sucrose;GO:0034285 response to disaccharide;GO:0006096 glycolytic process
FBgn0038952	CG7069	GO:0006096 glycolytic process;GO:0006757 ATP generation from ADP;GO:0009135 purine nucleoside diphosphate metabolic process
FBgn0003118	pnt	GO:0007429 secondary branching, open tracheal system;GO:0009997 negative regulation of cardioblast cell fate specification;GO:0051892 negative regulation of cardioblast differentiation
FBgn0264326	DNApol-epsilon255	GO:0006287 base-excision repair, gap-filling;GO:0090592 DNA synthesis involved in DNA replication;GO:0006297 nucleotide-excision repair, DNA gap filling
FBgn0053111	CG33111	NA

FBgn0085384	CG34355	NA
FBgn0260634	eIF4G2	GO:0007140 male meiotic nuclear division;GO:0140013 meiotic nuclear division;GO:1903046 meiotic cell cycle process
FBgn0000039	nAChRalpha2	GO:0007271 synaptic transmission, cholinergic;GO:0042391 regulation of membrane potential;GO:0098655 cation transmembrane transport
FBgn0262389	mir-1017	NA
FBgn0263490	mld	GO:0006697 ecdysone biosynthetic process;GO:0008205 ecdysone metabolic process;GO:1902653 secondary alcohol biosynthetic process
FBgn0039212	Syx18	GO:0006890 retrograde vesicle-mediated transport, Golgi to endoplasmic reticulum;GO:0007274 neuromuscular synaptic transmission;GO:0048193 Golgi vesicle transport
FBgn0039213	atl	GO:0016320 endoplasmic reticulum membrane fusion;GO:0048677 axon extension involved in regeneration;GO:0048686 regulation of sprouting of injured axon
FBgn0046685	Wsck	GO:0045087 innate immune response;GO:0007169 transmembrane receptor protein tyrosine kinase signaling pathway;GO:0007167 enzyme linked receptor protein signaling pathway
FBgn0039215	CG6695	NA
FBgn0051126	CG31126	NA
FBgn0020018	Ppox	GO:0006779 porphyrin-containing compound biosynthetic process;GO:0033014 tetrapyrrole biosynthetic process;GO:0006783 heme biosynthetic process
FBgn0003429	slo	GO:1900074 negative regulation of neuromuscular synaptic transmission;GO:0045433 male courtship behavior, veined wing generated song production;GO:0016545 male courtship behavior, veined wing vibration
FBgn0039324	CG10553	NA
FBgn0039325	CG10560	GO:0008150 biological_process
FBgn0039326	CG10562	NA
FBgn0266255	lncRNA:CR44950	NA
FBgn0051323	CG31323	NA

FBgn0053970	CG33970	GO:0055085 transmembrane transport;GO:0006810 transport;GO:0051234 establishment of localization
FBgn0263289	scrib	GO:0062237 protein localization to postsynapse;GO:0098877 neurotransmitter receptor transport to plasma membrane;GO:0098887 neurotransmitter receptor transport, endosome to postsynaptic membrane
FBgn0261618	larp	GO:0090296 regulation of mitochondrial DNA replication;GO:0090297 positive regulation of mitochondrial DNA replication;GO:2000767 positive regulation of cytoplasmic translation
FBgn0013813	Dhc98D	GO:0007018 microtubule-based movement;GO:0007017 microtubule-based process;GO:0006928 movement of cell or subcellular component
FBgn0015221	Fer2LCH	GO:0098711 iron ion import across plasma membrane;GO:1990461 detoxification of iron ion;GO:0033212 iron import into cell
FBgn0039863	CG1815	GO:0006355 regulation of transcription, DNA-templated;GO:1903506 regulation of nucleic acid-templated transcription;GO:2001141 regulation of RNA biosynthetic process
FBgn0039864	CG11550	NA
FBgn0264900	lncRNA:CR44091	NA
FBgn0039883	RhoGAP100F	GO:0048789 cytoskeletal matrix organization at active zone;GO:0048790 maintenance of presynaptic active zone structure;GO:0099558 maintenance of synapse structure
FBgn0024366	CG11409	NA
FBgn0283741	prage	GO:0007343 egg activation;GO:0001775 cell activation;GO:0007338 single fertilization
FBgn0262178	mir-981	NA
FBgn0052806	CG32806	GO:0008150 biological_process
FBgn0004647	N	GO:2000048 negative regulation of cell-cell adhesion mediated by cadherin;GO:0048627 myoblast development;GO:0006110 regulation of glycolytic process
FBgn0266350	lncRNA:CR44999	NA
FBgn0029657	CG12535	NA
FBgn0029658	CG14269	NA

FBgn0029663	CG10804	GO:0035725 sodium ion transmembrane transport;GO:0006814 sodium ion transport;GO:0006836 neurotransmitter transport
FBgn0029664	CG10802	GO:0006419 alanyl-tRNA aminoacylation;GO:0006450 regulation of translational fidelity;GO:0006418 tRNA aminoacylation for protein translation
FBgn0029665	CG14270	GO:0045039 protein insertion into mitochondrial inner membrane;GO:0051204 protein insertion into mitochondrial membrane;GO:0090151 establishment of protein localization to mitochondrial membrane
FBgn0052791	DIP-alpha	GO:0050808 synapse organization;GO:0034330 cell junction organization;GO:0016043 cellular component organization
FBgn0052792	ppk8	GO:0035725 sodium ion transmembrane transport;GO:0006814 sodium ion transport;GO:0030001 metal ion transport
FBgn0029672	CG2875	GO:0042254 ribosome biogenesis;GO:0022613 ribonucleoprotein complex biogenesis;GO:0044085 cellular component biogenesis
FBgn0266429	AstA-R1	GO:0007218 neuropeptide signaling pathway;GO:0007186 G protein-coupled receptor signaling pathway;GO:0007165 signal transduction
FBgn0261383	IntS6	GO:0034472 snRNA 3'-end processing;GO:0016180 snRNA processing;GO:0016073 snRNA metabolic process
FBgn0029798	CG4078	GO:0090657 telomeric loop disassembly;GO:1904429 regulation of t-circle formation;GO:1904430 negative regulation of t-circle formation
FBgn0029831	CG5966	GO:0016042 lipid catabolic process;GO:0006629 lipid metabolic process;GO:1901575 organic substance catabolic process
FBgn0053664	CG33664	GO:0008150 biological_process
FBgn0029930	CG12541	NA
FBgn0265457	lncRNA:CR44357	NA
FBgn0029941	CG1677	GO:0050779 RNA destabilization;GO:0043487 regulation of RNA stability;GO:0006401 RNA catabolic process
FBgn0029504	CHES-1-like	GO:0031571 mitotic G1 DNA damage checkpoint signaling;GO:0044819 mitotic G1/S transition checkpoint signaling;GO:1902807 negative regulation of cell cycle G1/S phase transition
FBgn0261873	sdt	GO:2000395 regulation of ubiquitin-dependent endocytosis;GO:2000397 positive regulation of ubiquitin-dependent endocytosis;GO:0070830 bicellular tight junction assembly
FBgn0015519	nAChRalpha3	GO:0042391 regulation of membrane potential;GO:0007268 chemical synaptic transmission;GO:0098916 anterograde trans-synaptic signaling

FBgn0026411	Lim1	GO:0007479 leg disc proximal/distal pattern formation;GO:0007449 proximal/distal pattern formation, imaginal disc;GO:0035223 leg disc pattern formation
FBgn0052694	CG32694	NA
FBgn0028342	ATPsyndelta	GO:0015985 energy coupled proton transport, down electrochemical gradient;GO:0015986 ATP synthesis coupled proton transport;GO:0006754 ATP biosynthetic process
FBgn0030186	CG2962	GO:0045944 positive regulation of transcription by RNA polymerase II;GO:0045893 positive regulation of transcription, DNA-templated;GO:1902680 positive regulation of RNA biosynthetic process
FBgn0267189	asRNA:CR45629	NA
FBgn0040941	CG15308	NA
FBgn0259241	CG42339	NA
FBgn0003366	sev	GO:0038083 peptidyl-tyrosine autophosphorylation;GO:0060250 germ-line stem-cell niche homeostasis;GO:0018108 peptidyl-tyrosine phosphorylation
FBgn0263111	cac	GO:0010524 positive regulation of calcium ion transport into cytosol;GO:0010522 regulation of calcium ion transport into cytosol;GO:0016057 regulation of membrane potential in photoreceptor cell
FBgn0030412	Tomosyn	GO:0016082 synaptic vesicle priming;GO:0006893 Golgi to plasma membrane transport;GO:1900073 regulation of neuromuscular synaptic transmission
FBgn0262187	mir-970	NA
FBgn0011837	Tis11	GO:0061158 3'-UTR-mediated mRNA destabilization;GO:1900151 regulation of nuclear-transcribed mRNA catabolic process, deadenylation-dependent decay;GO:1900153 positive regulation of nuclear-transcribed mRNA catabolic process, deadenylation-dependent decay
FBgn0030447	CG2200	GO:0006508 proteolysis;GO:0019538 protein metabolic process;GO:1901564 organonitrogen compound metabolic process
FBgn0263005	CG43313	GO:0030206 chondroitin sulfate biosynthetic process;GO:0030204 chondroitin sulfate metabolic process;GO:1903510 mucopolysaccharide metabolic process
FBgn0030687	RpIIIC160	GO:0045945 positive regulation of transcription by RNA polymerase III;GO:0006359 regulation of transcription by RNA polymerase III;GO:0009304 tRNA transcription
FBgn0030688	CG8952	GO:0006508 proteolysis;GO:0019538 protein metabolic process;GO:1901564 organonitrogen compound metabolic process
FBgn0030701	CG16952	GO:0061138 morphogenesis of a branching epithelium;GO:0001763 morphogenesis of a branching structure;GO:0002009 morphogenesis of an epithelium

FBgn0260938	tay	GO:0007633 pattern orientation;GO:0007628 adult walking behavior;GO:0090659 walking behavior
FBgn0030809	Ubr1	GO:0071596 ubiquitin-dependent protein catabolic process via the N-end rule pathway;GO:0016567 protein ubiquitination;GO:0043161 proteasome-mediated ubiquitin-dependent protein catabolic process
FBgn0030840	p-cup	GO:0008150 biological_process
FBgn0030841	CG8568	GO:0008150 biological_process
FBgn0085387	shakB	GO:0010644 cell communication by electrical coupling;GO:0016264 gap junction assembly;GO:0003254 regulation of membrane depolarization
FBgn0031145	Ntf-2	GO:0002807 positive regulation of antimicrobial peptide biosynthetic process;GO:0002777 antimicrobial peptide biosynthetic process;GO:0002805 regulation of antimicrobial peptide biosynthetic process

Supplementary Table S4. The top 20 enriched GO clusters with their represenative GO term. Count refers to the number of candidate genes in each cluster.

GO Term	Category	Description	Count	Log10(P)	Log10(q)
GO:0048666	GO Biological Processes	neuron development	55	-12.19	-8.2
GO:0002165	GO Biological Processes	instar larval or pupal development	40	-6.09	-3.43
GO:0042461	GO Biological Processes	photoreceptor cell development	14	-5.15	-2.62
GO:0007610	GO Biological Processes	behavior	39	-4.88	-2.39
GO:0040008	GO Biological Processes	regulation of growth	24	-4.69	-2.23
GO:0042692	GO Biological Processes	muscle cell differentiation	13	-4.69	-2.23
GO:0009719	GO Biological Processes	response to endogenous stimulus	21	-4.36	-1.95
GO:0008356	GO Biological Processes	asymmetric cell division	12	-4.23	-1.9
GO:0048056	GO Biological Processes	R3/R4 cell differentiation	5	-4.08	-1.8
GO:0007297	GO Biological Processes	ovarian follicle cell migration	14	-3.92	-1.68
GO:0001709	GO Biological Processes	cell fate determination	13	-3.9	-1.68
GO:0007611	GO Biological Processes	learning or memory	15	-3.8	-1.65
GO:0006096	GO Biological Processes	glycolytic process	6	-3.8	-1.65
GO:0030516	GO Biological Processes	regulation of axon extension	6	-3.8	-1.65
GO:0051254	GO Biological Processes	positive regulation of RNA metabolic process	30	-3.7	-1.58
GO:0016322	GO Biological Processes	neuron remodeling	8	-3.58	-1.51
GO:0007623	GO Biological Processes	circadian rhythm	14	-3.52	-1.47
GO:0060581	GO Biological Processes	cell fate commitment involved in pattern specification	6	-3.24	-1.3
GO:0090162	GO Biological Processes	establishment of epithelial cell polarity	4	-3.18	-1.25
R-DME-9675108	Reactome Gene Sets	Nervous system development	11	-3.14	-1.21
